# Global Perspectives on Immunization Against SARS-CoV-2 During Pregnancy and Priorities for Future Research: An International Consensus Paper From the World Association of Infectious Diseases and Immunological Disorders

**DOI:** 10.3389/fimmu.2021.808064

**Published:** 2021-12-23

**Authors:** Bahaa Abu-Raya, Shabir A. Madhi, Saad B. Omer, Gayatri Amirthalingam, Michelle L. Giles, Katie L. Flanagan, Petra Zimmermann, Miguel O’Ryan, Marco A. Safadi, Vassiliki Papaevangelou, Kirsten Maertens, Nasamon Wanlapakorn, Vicens Diaz-Brito, Eline Tommelein, Susanna Esposito

**Affiliations:** ^1^ Department of Pediatrics, University of British Columbia, Vancouver, BC, Canada; ^2^ South African Medical Research Council, Vaccines and Infectious Diseases Analytics Research Unit, Faculty of Health Sciences, University of the Witwatersrand, Johannesburg, South Africa; ^3^ African Leadership in Vaccinology Expertise, Faculty of Health Sciences, University of the Witwatersrand, Johannesburg, South Africa; ^4^ Department of Internal Medicine (Infectious Diseases), Yale School of Medicine, New Haven, CT, United States; ^5^ Department of Epidemiology of Microbial Diseases, Yale School of Public Health, New Haven, CT, United States; ^6^ Immunisation and Countermeasures Division, National Infection Service, Public Health England, London, United Kingdom; ^7^ Department of Obstetrics and Gynaecology, Monash University, Melbourne, VIC, Australia; ^8^ Department of Infectious Diseases, Peter Doherty Institute for Infection and Immunity, Melbourne, VIC, Australia; ^9^ School of Medicine, Faculty of Health Sciences, University of Tasmania, Launceston, TAS, Australia; ^10^ School of Health and Biomedical Science, Royal Melbourne Institute of Technology (RMIT) University, Melbourne, VIC, Australia; ^11^ Department of Immunology and Pathology, Monash University, Melbourne, VIC, Australia; ^12^ Tasmanian Vaccine Trial Centre, Clifford Craig Foundation, Launceston General Hospital, Launceston, TAS, Australia; ^13^ Faculty of Science and Medicine, University of Fribourg, Fribourg, Switzerland; ^14^ Department of Paediatrics, Fribourg Hospital HFR, Fribourg, Switzerland; ^15^ Microbiology and Mycology Program, Institute of Biomedical Sciences and Millennium Institute of Immunology and Immunotherapy, Faculty of Medicine, University of Chile, Santiago de Chile, Chile; ^16^ Department of Pediatrics, Santa Casa de Sao Paulo School of Medical Sciences, Sao Paulo, Brazil; ^17^ National and Kapodistrian University of Athens, Third Department of Pediatrics, University Hospital Attikon, Athens, Greece; ^18^ Centre for the Evaluation of Vaccination, Vaccine & Infectious Diseases Institute, Faculty of Medicine and Health Sciences, University of Antwerp, Antwerp, Belgium; ^19^ Center of Excellence in Clinical Virology, Department of Pediatrics, Faculty of Medicine, Chulalongkorn University, Bangkok, Thailand; ^20^ Department of Infectious Diseases, Parc Sanitari Sant Joan de Déu,, Barcelona, Spain; ^21^ Department of Pharmacy, Faculty of Medicine and Pharmacy, Vrije Universiteit Brussel, Brussels, Belgium; ^22^ Pediatric Clinic, Pietro Barilla Children’s Hospital, Department of Medicine and Surgery, University of Parma, Parma, Italy

**Keywords:** maternal immunization, pregnant women, SARS-CoV-2, COVID-19, maternal vaccination program

## Abstract

Severe acute respiratory syndrome coronavirus 2 (SARS-CoV-2) infection in pregnancy is associated with a higher risk for severe morbidity and mortality when compared with infection in non-pregnant women of childbearing age. An increasing number of countries recommend immunization against SARS-CoV-2 in pregnant women. Recent studies provide preliminary and supportive evidence on safety, immunogenicity and effectiveness of coronavirus disease 2019 (COVID-19) vaccines in pregnant women; however, important knowledge gaps remain which warrant further studies. This collaborative consensus paper provides a review of the current literature on COVID-19 vaccines in pregnant women, identifies knowledge gaps and outlines priorities for future research to optimize protection against SARS-CoV-2 in the pregnant women and their infants.

## 1 Introduction

Women infected with severe acute respiratory syndrome coronavirus 2 (SARS-CoV-2) when pregnant are at increased risk for severe morbidity and mortality compared with non-pregnant women of childbearing age (1–3). Data from the US Centers for Disease Control and Prevention (CDC) have shown that women of childbearing age who were pregnant were three times more likely to be admitted to the intensive care unit (ICU) (10.5 *vs*. 3.9 per 1,000 cases), 2.9 times more likely to require invasive ventilation (2.9 *vs*. 1.1 per 1,000 cases), 2.4 times more likely to require extracorporeal membrane oxygenation (0.7 *vs*. 0.3 per 1,000 cases), and 1.7 times more likely to die (1.5 *vs*. 1.2 per 1,000 cases) compared with non-pregnant women and after adjusting for age, ethnicity, and underlying medical conditions (1). Most neonates infected with SARS-CoV-2 are asymptomatic or have mild symptoms (4, 5).

Immunization of pregnant women induces humoral and cellular antigen-specific immune responses in the pregnant women. Vaccine-induced antibodies are transferred transplacentally to the fetus, and to the newborn after delivery *via* breastmilk. Immunization during pregnancy aims to protect the women against clinically relevant infection, reduce adverse infection related fetal outcomes and protect the infant during their first 4-6 months of life. Given the high risk of COVID-19 morbidity and mortality in pregnant women, several COVID-19 vaccines have been approved for use in this population in many countries. The World Health Organization (WHO) recommends immunization against SARC-CoV-2 in pregnant women when the benefits of immunization outweigh the potential risks of vaccines (6, 7). The US CDC currently recommends that pregnant women may choose to receive a COVID-19 vaccine (8). Universal immunization against SARS-CoV-2 in pregnancy is recommended in most (n=23) European countries (9).

To optimize the strategy of maternal immunization, we previously addressed critical knowledge gaps that need to be addressed in relation to vaccines against tetanus, pertussis, influenza, respiratory syncytial virus and group B *Streptococcus* (10). In the current consensus paper written by experts in infectious diseases, vaccinology and maternal immunization from different world regions, we summarize the current evidence in the field of COVID-19 vaccines in pregnant women, identify key knowledge gaps and priorities for future research strategies in this population.

## 2 Incidence and Burden of COVID-19 in Pregnant Women

There are limited data on the incidence of COVID-19 in pregnant women. Although some studies suggested higher rates of SARS-CoV-2 infection in pregnant women compared with adults of reproductive age, understanding the extent to which this reflects differences in exposure risk, threshold for investigating and rate of testing, rather than a genuine increase in susceptibility remains a challenge. Nevertheless, the growing evidence demonstrates an increased risk of severe adverse outcomes from SARS-CoV-2 infection in pregnant compared with non- pregnant women, particularly when infection occurs during late-second and early-third trimesters (11, 12). This increased burden of COVID-19 appears to be disproportionately affecting mother-neonate dyads from low- and middle- income countries (13). An increased risk of severe COVID-19 outcomes in pregnant women is likely attributable to several factors associated with pregnancy including immunological changes leading to dampening of cellular immunity (14), physiological changes such as reduced lung capacity and increased risk of thromboembolic disease in late pregnancy (1). Sociodemographic and clinical risk factors associated with poor outcomes have been identified through pregnancy cohorts worldwide. Women from Black, Asian and other ethnic minority backgrounds, maternal age >35 years, women who are overweight or obese, those with a history of smoking and women with co-morbidities such as hypertension or diabetes are at increased risk of developing severe COVID-19 (11, 15–17).

A systematic review and meta-analysis of studies published until January 2021 found an increased risk of preeclampsia (Odd ratio [OR] 1.33, 95% CI 1.03-1.73), preterm birth (OR 1.82, 95% CI 1.38-2.39), and stillbirth (OR 2.11, 95% CI 1.14-3.90) among pregnant women with SARS-CoV-2 infection compared with those without SARS-CoV-2 infection (18). Those who develop severe disease have increased rates of admission to an intensive care unit (ICU), requiring invasive ventilation and preterm birth. A recently published retrospective cohort study of pregnant women delivering across 7 hospitals in the USA during the first and second COVID-19 waves, women with COVID-19 were 2.8 times more likely to deliver preterm (19.0%; OR 95% CI 1.92-3.88) compared with women with asymptomatic infection (8.8%) or without SARS-CoV-2 infection (7.1%) (19).

In a population-based cohort study of 673 pregnant women with SARS-CoV-2 infection from 36 hospitals across South Africa, 217 (32.2%) were admitted for COVID-19 and 106 required critical care (9). The maternal mortality rate was high among women admitted with SARS-CoV-2 infection (1.5 per 1,000 in pregnant women *vs*. 1.2 per 1,000 in non-pregnant women) and higher in women admitted primarily for COVID-19 illness, with tuberculosis being the only co-morbidity associated with admission (20).

## 3 Burden of COVID-19 in Neonates and Infants

Mother-to-child transmission of SARS-CoV-2 can occur either *in utero via* the placental route (21, 22), intra-partum or in the early postnatal period. While SARS-CoV-2 infection in pregnant women is associated with an increased risk of preterm delivery, fetal growth restriction, stillbirth, and neonatal admission to the ICU, it has not been associated with an increased risk of neonatal death (18, 23, 24). Recent evidence indicates that 2-5% of neonates born to mothers with COVID-19 are infected with SARS-CoV-2 (15, 25–27).

SARS-CoV-2 infection in neonates usually results in mild disease and a recent meta-analysis showed that 20% of neonatal infections were asymptomatic. Among symptomatic cases, 48% and 20% had mild and moderate signs of COVID-19, respectively (28). The most common clinical presentations of neonatal COVID-19 were respiratory distress (40%), fever (32%) and feeding intolerance (24%) (29). Although neonatal COVID-19 is generally a mild disease, infants under one year were reported to have more critical illness than older children (14% in <1 year *vs*. 5% in 0-18 years), making this age group relatively more vulnerable to COVID-19-associated morbidity than older children (30).

Based on the literature review and consultation among authors, a consensus on priorities for future research related to COVID-19 in pregnant women and infants was reached (**Table 1**).

**Table 1:** Consensus on priorities for future research related to COVID-19 in pregnant women and infants.

**COVID-19 in pregnant women**
Placental pathology associated with SARS-CoV-2 infection at varying stages of pregnancy.
The pathogenesis of SARS-CoV-2 infection at different time points in pregnancy, time-dependent risk of infection and resultant adverse fetal outcomes.
Rates of SARS-CoV-2 infection in pregnant and non-pregnant women with similar exposure risks.
The clinical course of SARS-CoV-2 infection, including symptomatic and asymptomatic infection, in pregnant women.
Long-term effect of COVID-19 in the women infected by SARS-CoV-2 during pregnancy.
**COVID-19 in infants**
Risk factors for maternal transmission and neonatal susceptibility to SARS-CoV-2 infection.
Long-term follow up on physical growth, development, and COVID-19-related complications among infants infected with SARS-CoV-2 and long-term follow up of infants exposed to SARS-CoV-2 *in utero*.

## 4 Transfer of Anti-SARS-CoV-2 Antibodies Following Infection in Pregnancy

Anti-SARS-CoV-2 IgG antibodies have been detected in cord blood of women infected with SARS-CoV-2 during gestation with variable efficiency of transplacental transfer of these antibodies to the fetus (31–34). Among 37 women with positive SARS-CoV-2 PCR during pregnancy, with a median time from infection to delivery of 4 weeks, 24 and 26 women had detectable anti-spike (S) protein receptor binding domain (RBD) and anti-nucleocapsid (N) IgG, respectively (31). The mean transplacental transfer ratio (defined as ratio of IgG levels in cord/neonatal blood compared with maternal levels) of anti-RBD and anti-N IgG in the mother-newborn dyad were 0.82 (n=20) and 0.85 (n=22), respectively. The transplacental transfer ratio was low (0.25-0.27) in 4 preterm infants born at <37 weeks gestational age (31). In another study, the transplacental transfer ratio of anti-RBD IgG was 0.9 in pregnant women who were SARS-CoV-2 IgG- and/or IgM- seropositive at delivery (n=83), and 54% of those tested (44/82) were positive by SARS-CoV-2 PCR during pregnancy (32). Similarly, in another cohort of 22 pregnant women with antenatal SARS-CoV-2 infection at a mean of 30 days prior to delivery, transplacental transfer for anti-RBD, -N and -S IgG was <1 (33). Furthermore, the transplacental transfer ratio was <1 for antibodies mediating cellular phagocytosis, neutrophil phagocytosis and complement disposition against SARS-CoV-2 (33). Moreover, the ratio of anti-SARS-CoV-2 IgG transfer was confirmed in the same study in a subset of women infected during the third trimester, in whom the time of delivery relative to ante-natal infection (range 10-50 days) was not associated with difference in the transplacental ratio of transfer (33). However, an anti-SARS-CoV-2 IgG transfer ratio >1 was reported in women infected during the second trimester of their pregnancy (33). In another study, transplacental transfer ratios of IgG to S1, S2, RBD, and N antigens were significantly higher in pregnant women who were infected by SARS-CoV-2 prior to 30 weeks gestation compared with those infected after 30 week gestation (34). Collectively, these limited data suggest that timing of maternal infection with SARS-CoV-2 in pregnancy impacts on efficiency of transplacental transfer of anti-SARS-CoV-2 antibodies with third-trimester infection associated with less efficient transfer of SARS-CoV-2-specific antibodies while infection earlier in pregnancy is associated with higher transfer of maternal antibodies, as reported for antibodies induced by other vaccine antigens (35–39). Nevertheless, since most studies either did not report the timing of infection in pregnancy or included women who had infection close to delivery, it is challenging to conclude on the precise effect of timing of infection on efficiency of anti-SARS-CoV-2 IgG transplacental transfer. Also, the clinical relevance of these antibodies is difficult to interpret as no correlate of protection has yet been identified for COVID-19, however, it is likely that these antibodies could provide some clinical protection (40, 41).

### 4.1 Fetal Immune Response to Intrauterine Exposure to SARS-CoV-2

Anti-SARS-CoV-2 IgM antibodies have been detected in cord blood samples of women infected with SARS-CoV-2 during pregnancy, suggesting intrauterine vertical transmission of SARS-CoV-2 and fetal immune response. In one study, anti-SARS-CoV-2 IgM was detected at birth in two out of six newborns of women infected with SARS-CoV-2 during pregnancy (42), and in a 2-hour old newborn (43). In addition, anti-SARS-CoV-2 IgM was detected in one out of 31 cord blood samples of women infected with SARS-CoV-2 within 1-17 days prior to delivery (44), and in a 29-week preterm infant (45). Among 65 women with positive SARS-CoV-2 PCR detected during various stages in pregnancy (10-38 weeks), five neonates had anti-SARS-CoV-2 IgM in cord blood, with none of the newborns exhibiting signs of COVID-19 (34). The clinical relevance of exposure to maternal SARS-CoV-2 antigens *in utero* (21, 22, 46), leading to congenital infection, requires further assessment. One case report reported fetal death after maternal infection with SARS-CoV-2 during early third trimester of pregnancy (22). Another case report reported a late preterm infant (35 weeks gestation) born to a mother infected during the third trimester of pregnancy who presented with neurological symptoms with full recovery upon follow up (23). A systematic review reported a proportion of 3.2% (22/936) of vertical transmission following maternal infection in the third trimester of pregnancy after combing data from 38 cohort or case series studies (47). However, no studies reported on vertical transmission following maternal infection during early pregnancy (47).

Although a recent systematic review reported that maternal infection with SARS-CoV-2 was associated with higher risk of preterm delivery and stillbirth (18), the proportion of these fetuses with congenital SARS-CoV-2 infection is unknown. Thus, large studies are needed to assess the short and long-term effects of *in utero* exposure to SARS-CoV-2.

## 5 Transfer of SARS-CoV-2 and Anti- SARS-CoV-2 Antibodies in Breast Milk Following Infection in Pregnancy

Concerns have been raised about whether SARS-CoV-2 is shed in breastmilk of infected mothers and can be transmitted to their breastfeeding infant. While several early small studies found no evidence of SARS-CoV-2 in breastmilk, it was reported early in the pandemic that one of two women infected by SARS-CoV-2 had detectable SARS-CoV-2 RNA in her breastmilk for four consecutive days (48). The mother followed strict safety precautions and wore a surgical mask whilst handling her child, but the infant tested positive for SARS-CoV-2 at the time that the virus RNA was first detected in breastmilk, raising the possibility that breastmilk was the source of infection, however another mode of infection could not be excluded. A systematic review of 50 articles found that 12 of 183 women (5% [95% CI 2-15%]) with confirmed SARS-CoV-2 infection across 48 studies had the SARS-CoV-2 genome present in their breastmilk (49). Half of the 12 neonates from these women tested positive for SARS-CoV-2 by nasopharyngeal swab (6/12) and 33% (4/12) were symptomatic of whom one required respiratory support. A US study analyzing consecutive breastmilk samples from 18 women with SARS-CoV-2 infection reported that one of 64 samples had detectable SARS-CoV-2 RNA (50) which was negative for replication competent virus by culture assay. Another study of 110 women similarly found no evidence of infectious virus in breastmilk by culture despite 6% being reactive on PCR testing (47).

The aforementioned systematic review summarized ten studies, showing that 83% (95% CI 32-98%) of 89 women had anti-SARS-CoV-2 antibodies in their breastmilk, mostly with IgA (49). Another study similarly found that 76% and 80% of breast milk samples from 18 women infected with SARS-CoV-2 had anti-SARS-CoV-2 IgA and IgG, respectively (51). Importantly, 62% of these samples were capable of neutralizing SARS-CoV-2 infectivity *in vitro*. Overall, these studies suggest that a very low percentage of lactating women infected with SARS-CoV-2 have detectable SARS-CoV-2 RNA in their breastmilk, and that detected virus may not be replication competent or pose a risk of infection to their neonates. Since the breastmilk of most women infected with SARS-CoV-2 contains specific IgA and/or IgG, the protective benefits of breastfeeding may outweigh the risk of infection *via* breastmilk. In line with the WHO and CDC recommendations, initiation and continuation of breastfeeding should be recommended for women with COVID-19 (52, 53).

Based on the literature review and consultation among authors, a consensus on priorities for future research related to transfer of anti-SARS-CoV-2 antibodies after SARS-CoV-2 infection in pregnancy across the placenta and in breastmilk was reached (**Table 2**).

**Table 2:** Consensus on priorities for future research related to transfer of anti-SARS-CoV-2 antibodies after SARS-CoV-2 infection in pregnancy across the placenta and in breastmilk.

**Transplacental transfer**
Effect of timing of infection with SARS-CoV-2 in pregnancy on efficiency of transfer of maternal antibodies across the placenta, especially for infections early in pregnancy
Kinetics of anti-SARS-CoV-2 antibodies transplacentally transferred to infants.
Function of anti-SARS-CoV-2 antibodies transplacentally transferred to infants.
Correlate(s) of protection and cut-off of protection of anti-SARS-CoV-2 antibodies transplacentally transferred to infants from clinical disease in infancy.
Clinical relevance of anti-SARS-CoV-2 antibodies transplacentally transferred to infants.
**Transfer *via* breastmilk**
Large prospective study to evaluate the risk of SARS-CoV-2 infection versus protection among breastfeeding children of lactating women, infected with SARS-CoV-2.
Humoral immune responses in breastmilk of lactating women, infected with SARS-CoV-2 (titer, subclass, glycosylation profile and functional properties of infection-induced antibodies, including neutralization properties).
Impact of COVID-19 infection on cellular immunity in breastmilk: 1. T-cell subsets directed against SARS-CoV-2 expressed in breastmilk after COVID-19 infection. 2. T-cell dependent cytokine production observed in breastmilk after COVID-19 infection. 3. T-cell help relation to the B-cell antibody production after COVID-19 infection?

## 6 Immunization During Pregnancy

### 6.1 Ethics of Immunization Against COVID-19 in Pregnancy

Immunizing pregnant women requires nuanced ethical considerations – COVID-19 vaccines are no different. In fact, the need for ethical decision-making is even more acute in a pandemic as information regarding risk and benefits is evolving. There are three major ethical questions related to COVID-19 vaccines in pregnancy. First, inclusion of pregnant women in COVID-19 vaccine trials. For many years, vaccine companies and even some independent investigators have taken a “risk averse” approach to inclusion of pregnant women in vaccine trials, and they have been excluded. However, in recent years, there has been an increasing ethical and scientific consensus to include pregnant women in early human trials of vaccines with potential use in pregnancy (54). Unfortunately, for COVID-19 vaccines, pregnant women were not included in any pre-licensure trials and most vaccine companies had an unclear clinical development plan which led to confusion and concern among pregnant women and decision-making with limited data. The second major ethical question is whether pregnant women should receive a COVID-19 vaccine? With increasing evidence of safety of various COVID-19 vaccines in pregnancy, and with increasing understanding of the disproportionate negative impact of COVID-19 on pregnant women, it is recommended and ethically appropriate for pregnant women to receive a COVID-19 vaccine when the available data suggest reasonable efficacy and safety. The third major ethical question is whether is how much priority should be given to pregnant women compared to other high-risk groups?. The COVID-19 vaccine working group of the WHO Strategic Advisory Group of Experts included pregnant women in the Stage II of its Vaccine Prioritization Roadmap, as part of the “Groups with comorbidities or health states determined to be at significantly higher risk of severe disease or death” (55). Maternal immunization during pregnancy is an active area of research. Given that SARS-CoV-2 is a novel human virus with recently developed vaccines, the need for research on COVID-19 vaccines is more acute.

### 6.2 Safety

Most safety data for COVID-19 vaccines in pregnancy has come from ‘real world’ evidence gathered after regulatory approval and implementation rather than from the initial clinical trials, where pregnant women were largely excluded. Furthermore, most currently published safety data in pregnancy are for mRNA vaccines including BNT162b2 and mRNA-1273 vaccines. One of the first and largest studies reporting safety data in pregnant women was published from the US and included over 35,000 pregnant women had received an mRNA COVID-19 vaccine (56). The rate of reported side effects was similar to non-pregnant women, with pain at the injection site reported slightly more often, but systemic symptoms such as fever and tiredness reported less often (56). This study also reported the outcomes for 827 completed pregnancies (56). Adverse pregnancy outcomes such as preterm delivery, stillbirth, small for gestational age infants and congenital anomalies occurred at a similar rate to the background rate in the general population (56). The data that informed this publication are part of the V-safe COVID-19 Vaccine Pregnancy Registry, a smartphone-based tool that uses text messaging and web surveys to provide personalized check in after receipt of a COVID-19 vaccine (57). This pool of data is continuing to accumulate safety information over time and was presented at the US CDC Advisory Committee on Immunization Practices meeting on September 22, 2021. The additional safety data presented confirmed earlier reports that there is no increased rate of adverse pregnancy outcomes compared to background rates (58). The findings from this large study are supported by other smaller studies (59–62). In addition, animal studies of BNT162b2 and mRNA-1273 vaccines have not shown any negative effects on fertility or pregnancy (63, 64).

The effect of timing of immunization during pregnancy and safety are critical considerations in deciding the recommended strategy of immunization during pregnancy, and it is particularly important to determine whether immunization during early pregnancy is associated with spontaneous abortion or birth defects. Adverse events following immunization against SARS-CoV-2 at different time points in pregnancy have been found to be comparable to those in non-pregnant women regardless of their gestational age (56, 65, 66). While pregnant women that were studied were immunized from the peri-conception period to the third trimester, data available on neonatal outcomes from women vaccinated in early pregnancy are limited (56). Data from the US CDC including almost 2,500 women revealed that the cumulative risk of spontaneous abortion in women immunized against COVID-19 between 6 to < 20 weeks of gestation was 14.1% and not different from that reported in historical cohorts (67). Additionally, in a retrospective study on safety of COVID-19 immunization among pregnant women, the rate of major congenital anomalies was 2.2%, consistent with that expected without immunization. In other studies, no specific pattern of congenital anomalies was observed, although none of the mothers had received COVID-19 vaccine in the first trimester or in the periconception period (56, 68).

Overall, the data on safety of COVID-19 vaccines in pregnancy are growing but still have some limitations, especially for several vaccine platforms such as vector-based and inactivated virus vaccines. In addition, more safety data on immunization during the first trimester are needed. In addition to “real world” data, information from clinical trials will also be available in the future as BNT162b2 vaccine is being studied in a global clinical trial in pregnant women (69), with hopefully other companies following suit.

Based on the literature review and consultation among authors, a consensus on priorities for future research related to ethics and safety of immunization against SARS-CoV-2 in pregnancy was reached (**Table 3**).

**Table 3:** Consensus on priorities for future research related to ethics and safety of immunization against SARS-CoV-2 in pregnancy.

Acceptance of COVID-19 vaccines and strategies to increase it.
First trimester immunization and risk of spontaneous abortion or congenital abnormalities.
Adverse events and congenital anomalies by timing of immunization during pregnancy.
Safety in setting of co-administration with other vaccines routinely offered in pregnancy (i.e. influenza, tetanus, diphtheria and acellular pertussis vaccines).

### 6.3 Immunogenicity

Few studies have investigated the immunogenicity of COVID-19 vaccines in pregnant women (34, 60, 66, 70, 71). Those that have been published included less than 100 pregnant women and either used the BNT162b2 and/or mRNA-1273 vaccines (34, 60, 66, 70, 71). Antibodies against RBD were measured in all studies (34, 60, 66, 70, 71), while some studies additionally measured antibodies against S1 and S2 proteins (34, 60, 70). Only one study investigated T cell responses (60). Three studies each compared antibody levels between immunized pregnant and non-pregnant women (60, 66, 70) or between immunized and SARS-CoV-2 infected pregnant women, respectively (34, 60, 70). One study included only immunized pregnant women without a control group (71). In four of the studies antibodies in cord blood were measured (34, 60, 70, 71), confirming that antibodies elicited by SARS-CoV-2 mRNA immunization during pregnancy are capable of crossing the placenta. Specifically, both S- and RBD- IgG antibodies as well as neutralizing antibodies are transplacentally transferred to the newborns at birth. After maternal immunization, two studies reported similar antibody levels in maternal and cord blood (34, 71), while two other studies reported lower levels of total SARS-CoV-2 specific antibodies and neutralizing antibodies in cord blood (60, 70). The latter findings contrast with the transplacental transfer of other antibodies after maternal immunization, for example pertussis, where levels in cord blood are usually higher than maternal levels (72). The transplacental transfer ratio for anti- RBD, S1 and S2 IgG between vaccinated and infected women was similar (34).

#### 6.3.1 Antibody Immune Response in Pregnant *vs*. Non Pregnant Women

In all but one study (66), SARS-CoV-2 specific antibody levels after immunization were similar in pregnant and non-pregnant women (60, 70, 71). The antibody levels were higher after vaccination than after natural infection, except for antibodies against the N protein, which is not an included antigen in the use in COVID-19 vaccines used (34, 60, 70). Although one study showed that pregnant women have significantly lower SARS-CoV-2 IgG levels after immunization with the BNT162b2 mRNA vaccine compared with non-pregnant women (66), other studies showed comparable humoral immune responses between pregnant and non-pregnant women for BNT162b2 and mRNA-1273 (59, 60). In one study, no differences in binding, neutralizing and functional non-neutralizing antibody responses were found between pregnant and non-pregnant women after mRNA COVID-19 immunization (60).

#### 6.3.2 Number of Doses and Timing of Immunization During Pregnancy

Currently the magnitude of transplacental antibody transfer following a single dose of COVID vaccine remains uncertain. One dose of BNT162b2 vaccine led to a rapid increase in SARS-CoV-2 specific IgG levels in pregnant women, and 14 days after the first vaccine dose, anti-SARS-CoV-2 antibodies were detected in cord blood (34). However, another study showed that anti-SARS-CoV-2 antibodies could not be found in cord blood if the first vaccine dose (82% received either BNT162b2 or mRNA-1273 vaccine) was given to women less than 3 weeks before delivery (71). Two maternal SARS-CoV-2 vaccine doses in pregnancy led to significantly higher maternal and cord anti-SARS-CoV-2 IgG levels compared to one dose (34, 70, 71), but not of IgA levels (34, 70). One study reported higher IgA responses against RBD and S protein after immunization with mRNA-1273 compared to immunization with BNT161b2 vaccine, which was thought to be attributed to the extended interval between the two doses for the former vaccine (70).

The timing of immunization during pregnancy is important for optimal protection provided for pregnant women and their infants. A recent study indicated that the earliest detection of anti-SARS-CoV-2 antibodies in cord blood occurs 16 days post first immunization while the placental transfer ratio correlates with the number of weeks elapsed post second vaccine dose (range 0-10 weeks) (73). Similarly, anti-S antibody levels in cord blood were found to be positively correlated with time between immunization and delivery (74).

Clinical trials investigating the immunogenicity of the BNT161b2 and the Ad26.COV2.S vaccine in pregnancy are currently ongoing (75–77). Another clinical trial investigating the effect of two different time intervals (4 to 6 *vs*. 8 to 12 weeks) on the immunogenicity of the mRNA-1273 and BNT162b2 vaccines in pregnancy is also currently recruiting participants (78).

#### 6.3.3 Induction of Vaccine-Induced Antibodies in Breast Milk

Transfer of passive and active immunity through human milk is a key element in infant protection against infections. To date, several prospective cohort studies have shown presence of antibodies (mainly IgA and IgG) in breast milk of lactating mothers immunized against SARS-CoV-2, either with mRNA (79–83), or adenoviral vaccines (84). These studies showed that antibody levels in breast milk increase after the second dose and are positively associated with maternal serum levels (79–83). Moreover, one study that evaluated the immunogenicity of mRNA vaccines also revealed the presence of binding and neutralizing antibodies in breast milk (60). One study including lactating women vaccinated with the inactivated virus COVID-19 vaccine found detectable anti-SARS-CoV-2 IgA antibodies in the breast milk (85). These data could indicate that breastfeeding in infancy has the potential to add to the protection conferred to infants *via* antibodies transferred across the placenta. In addition, one study showed that no mRNA is transferred *via* breastmilk (86).

#### 6.3.4 Cellular Immune Response

In a small single study, immunization with mRNA-1273 or BNT162b2 vaccines in pregnancy, during lactation and in non-pregnant women was associated with comparable percentage of spike-specific IFN-γ production by CD4 T cells, CD4 central memory T cells, CD8 T cells, and CD8 central memory T cells (60). However, there are currently no data on production of other cytokines or the effect on innate immune system.

Based on the literature review and consultation among authors, a consensus on priorities for future research related to immunogenicity of immunization against SARS-CoV-2 in pregnancy was reached (**Table 4** **)**.

**Table 4:** Consensus on priorities for future research related to immunogenicity of immunization against SARS-CoV-2 in pregnancy.

**Antibody immune response**
Immunogenicity in pregnant women of all licensed COVID-19 vaccines.
Vaccine-induced antibody transfer kinetics across all pregnancy trimesters.
The impact of booster COVID-19 immunization (following completion of 2 doses of primary immunization) in pregnancy.
Co-administration with other vaccines given in pregnancy and immune response to COVID-19 vaccines
**Number of doses and timing of immunization**
Optimal timing of immunization during pregnancy and the ideal time interval between the two doses.
Immune response of heterologous schedules with different number of vaccine does
**Induction of antibodies in breast milk**
Protection against SARS-CoV-2 in breastfeeding infants of vaccinated women and it’s duration.
Correlate(s) of protection of vaccine-induced anti-SARS-CoV-2 antibodies in breast milk.
Duration of humoral protection provided *via* breastfeeding.
Studies on lactating women vaccinated with non-mRNA-based COVID-19 vaccines.
**Cellular immune response**
Magnitude and diversity of cellular immune responses (adaptive and innate) after immunization at different time point during pregnancy.

## 7 Real-World Vaccine Effectiveness

Although recognized as a subpopulation of increased risk for severe COVID-19, pregnant and lactating women were not included in phase 3 safety and efficacy clinical trials of the current available COVID-19 vaccines in use. However, after being recommended by advisory committees and regulatory agencies, COVID-19 vaccines, particularly the mRNA vaccines, are being extensively used worldwide in pregnant and lactating women, providing an opportunity to evaluate real-world data on the effectiveness of these vaccines against the different outcomes of COVID-19. A large retrospective population-based cohort study compared the risk of SARS-CoV-2 infection among pregnant women immunized with the BNT162b2 mRNA vaccine versus unimmunized pregnant women. Immunization was associated with a significantly reduced risk of SARS-CoV-2 infection, with an adjusted hazard ratio (HR) of 0.22 (95% CI, 0.11-0.43, *P*<0.001), corresponding to an estimate of vaccine effectiveness (1 − HR) of 78% (87).

Another observational cohort study, including pregnant women aged 16 years or older who were immunized between 20 December 2020 and 3 June 2021, naïve for SARS-CoV-2 infection, found that the BNT162b2 mRNA vaccine effectiveness from 7 through to 56 days after the second dose was 96% (95% CI, 89–100%) for any documented infection, 97% (95% CI, 91–100%) for symptomatic infections and 89% (95% CI, 43–100%) for COVID-19-related hospitalization (62). Additionally, data from a comprehensive vaccine registry, identifying information from healthcare systems in the US found that women who were immunized in pregnancy were less likely than unimmunized pregnant women to experience SARS-CoV-2 infection before delivery (2/140 [1.4%] *vs*. 210/1861 [11.3%], P<0.001) (88).

Although preliminary data suggest that mRNA COVID-19 vaccines are highly effective in pregnant women, with comparable effectiveness estimates that were reported in the general population, evidence is still limited to few observational studies. Based on the literature review and consultation among authors, a consensus on priorities for future research related to efficacy and effectiveness of immunization against SARS-CoV-2 in pregnancy was reached (**Table 5**).

**Table 5:** Consensus on priorities for future research related to efficacy and effectiveness of immunization against SARS-CoV-2 in pregnancy.

Vaccine effectiveness across different populations of pregnant women
Vaccine effectiveness against new variants of concern
Vaccine effectiveness studies with non-mRNA formulations (e.g. inactivated vaccines, non-replicating vectors).
Ideal timing of immunization and number of doses to provide protection to the pregnant women and their infants.

## 8 Consensus on Immunization During Pregnancy and Future Directions

Although pregnant women were not included in the initial COVID-19 vaccine trials, which led to insufficient data to make initial evidence-based recommendations, significant body of research has accumulated since early 2021 to support the recommendation of immunization against SARS-CoV-2 in pregnancy. With the limited information available, we recommend immunization of pregnant women with mRNA vaccines. This recommendation is based mainly on the increased risk for severe infection during pregnancy and the acceptable safety, immunogenicity and effectiveness profile of the mRNA vaccines.

Following review of the current literature and consultation amongst experts in the fields of infectious diseases, vaccination and immunization during pregnancy, several gaps in knowledge and priorities for research were identified and are proposed (**Tables 1**–**5**). Addressing these priorities in future research has the potential to increase our understanding in different aspects of immunization against SARS-CoV-2 in pregnancy and to optimize protection for both the mothers and their infants. To provide a better understanding of the safety, immunogenicity, and efficacy/effectiveness of different COVID-19 vaccines, several companies have commenced clinical trials in pregnant and lactating women. Pfizer is conducting a randomized, placebo-controlled, observer-blinded phase 2/3 study to evaluate the safety, tolerability, and immunogenicity of the BNT162be mRNA vaccine in healthy pregnant women (89). Janssen is conducting a phase 2 randomized, open-label study of its non-replicating adenoviral vaccine, Ad26.COV2.S, including pregnant women (90). Moderna is also evaluating in a prospective observational study, the potential impact of its mRNA vaccine, mRNA-1273, on pregnancy and birth outcomes (91). Based on the successful experience with other inactivated vaccines used during pregnancy, studies should be conducted to evaluate the safety, immunogenicity and effectiveness of the currently authorized COVID-19 inactivated vaccines in pregnant women. Uptake of COVID-19 vaccine in pregnancy varies between countries and settings but has been generally low. The reported uptake of ≥1 dose of COVID-19 vaccine in pregnancy was 15-23% in Scotland (92), 22% in the UK (93) and 16.3% in the US (94). Efforts should be made to increase vaccine uptake in pregnancy.

Unique to COVID-19 is that most current studies evaluated pregnant women who were likely naïve to SARS-CoV-2 prior to pregnancy. It is expected that the level of pre-existing immunity of pregnant women and their infants to SARS-CoV-2 will increase as the pandemic evolves further and the number of pregnant women who enter pregnancy with natural or vaccine-induced immunity increases (95). Ongoing evaluation of the gaps in knowledge as levels of pre-existing immunity in pregnancy women increases is important and poses a unique challenge for the COVID-19 pandemic and should be included in study design and interpretation of results to inform pregnant women, public health policy makers, clinicians and researchers. Finally, in future pandemics where pregnant women and/or young infants are at high risk for severe morbidity and mortality, pregnant women should be included early in early phases of clinical trials.

## Author Contributions

BA and SE proposed the project and coordinated the study group. BA, SM, SO, GA, MLG, KF, PZ, MS, VP, KM, VD-B, SE, MO’R, ET, and NW contributed to the writing of first draft of the consensus statements and revised the initial draft of the manuscript. BA, SM, SO, GA, MLG, KF, PZ, MS, VP, KM, VD-B, SE, MO’R, and ET reviewed and edited the manuscript, provided comments and suggested references and substantially contributed to the content of the manuscript. BA, SM, SO, GA, MLG, KF, PZ, MS, VP, KM, VD-B, SE, MO’R, and ET approved the final version of the manuscript. All authors contributed to the article and approved the submitted version.

## Funding

The publication of this manuscript was supported by the World Association for Infectious Diseases and Immunological Disorders (WAidid).

## Conflict of Interest

SE: Research support from GSK, Sanofi and Vifor. Speaker’s fees from GSK, Pfizer, Novartis, Sanofi Pasteur and MSD in the past three years. BA is supported by Michael Smith Health Research BC. MO’R: Received research funding for Phase III Covid-19 vaccine trial from Janssen; Received research funding for Phase II Pneumococcal conjugate vaccine trial from Pfizer. VD-B: no conflict of interest to declare. SM: Institution received funding on COVID-19 from BMGF, South African Medical Research Council and Novavax. Also, participating in clinical trials of Pfizer/Biontech and JJ Covid-19 vaccines in pregnant women. MS is a member of the data safety and monitoring board for Janssen and CEPI and received research grants and/or consultancy fees from Astra Zeneca, Janssen, Pfizer, Sanofi, Seqirus, Takeda, MSD and GSK. KF is a member of the Australian Technical Advisory Group on Immunisation noting that this paper represents her own personal view; received honoraria as a member of the vaccine advisory boards for Seqiris and Sanofi Pasteur in the last 5 years.

The remaining authors declare that the research was conducted in the absence of any commercial or financial relationships that could be construed as a potential conflict of interest.

## Publisher’s Note

All claims expressed in this article are solely those of the authors and do not necessarily represent those of their affiliated organizations, or those of the publisher, the editors and the reviewers. Any product that may be evaluated in this article, or claim that may be made by its manufacturer, is not guaranteed or endorsed by the publisher.
